# A Surgical Management of Mediastinal Parathyroid Adenoma: A Regional Experience in Malaysia

**DOI:** 10.7759/cureus.56792

**Published:** 2024-03-23

**Authors:** Yen Zhir Tay, Narendran Balasubbiah, Raflis Ruzairee Awang, Benedict Dharmaraj Retna Pandian, Narasimman Sathiamurthy

**Affiliations:** 1 General Surgery, Thoracic Surgery Unit, Kuala Lumpur Hospital, Kuala Lumpur, MYS; 2 General Surgery, Breast and Endocrine Surgery Unit, Kuala Lumpur Hospital, Kuala Lumpur, MYS

**Keywords:** video-assisted thoracoscopic surgery (vats), mediastinum, ectopic, parathyroid adenoma, primary hyperparathyroidism

## Abstract

Primary hyperparathyroidism (PHPT) usually presents with symptoms of hypercalcemia which is due to excessive secretion of parathyroid hormone (PTH). Surgical removal of the secreting tumor either adenoma or hyperplasia remains the mainstay of treatment. Around 2% to 25% of the lesions are located in the mediastinum. We reviewed our institution's surgical treatment and approach to mediastinal parathyroid adenoma (MPA).

We retrospectively reviewed the demography, comorbidities, clinical presentation, surgical approach, and outcome for patients in our institution who underwent surgery for MPA from September 2019 until August 2023. All patients with MPA who underwent surgery were included in the review. The surgical approaches used were both video-assisted thoracoscopic surgery (VATS) and median sternotomy.

There were three patients with PHPT due to MPA who underwent surgery. Out of the three patients, two were female. The mean age was 48.6 years old, ranging from 16 to 66 years old. All of them presented with PHPT with a raised mean serum calcium level of 3.52 mmol/L (range: 2.84-4.38 mmol/L) and a mean PTH or intact PTH (iPTH) level of 274.6 pmol/L (range: 8.87-695 pmol/L). Ultrasound of the neck was performed for all the patients before further investigations were done to look for the ectopic parathyroid gland. Computed tomography (CT) of the thorax showed mediastinal parathyroid mass in all the patients with an average size of 2.4 x 2.1 x 2.3cm (range: 1.3-3.8cm), which showed uptake in 99mTc-hexakis-2-methoxyisobuthylisonitrile (Tc99m-MIBI) scintigraphy. VATS was performed for two cases and an upper partial sternotomy was performed for one patient. Postoperatively, iPTH and serum calcium levels were reduced significantly for all patients. There were no post-operative complications in our study.

Comprehensive diagnostic imaging and surgical planning are important for the localization of MPA. In our review, all cases were promptly diagnosed and underwent surgery without complication.

## Introduction

Primary hyperparathyroidism (PHPT) may present with various symptoms secondary to hypercalcemia. It can be caused by parathyroid hyperplasia, parathyroid adenoma, or carcinoma. The incidence of ectopic parathyroid tumors is around 2% to 25% and it is commonly located in the neck or superior mediastinum region [1,2]. Surgical removal of the secreting tumor remains the mainstay of treatment. We reviewed the surgical treatment of mediastinal parathyroid adenoma (MPA) in our institution.

## Case presentation

We report on three subjects of MPA. All three patients with PHPT due to MPA underwent surgery in our institution from September 2019 until August 2023, within five years. We retrospectively examined the demographics, comorbidities, clinical presentation, surgical approach, and outcomes of these patients.

In our center, video-assisted thoracoscopic surgery (VATS) was performed under general anesthesia using a double-lumen endotracheal tube with single-lung ventilation. Patients are positioned in the left lateral decubitus position with a 4cm incision made over the right fourth intercostal spaces lateral to the anterior axillary line. Operations were performed without carbon dioxide (CO_2_) insufflation and we used a standard 10mm rigid thoracoscope with a 30-degree angle and other thoracoscopic instruments through the single opening overlying a wound protector.

For more accurate intraoperative localization, the parathyroid adenoma was exposed to near-infrared (NIR) light with a wavelength of 690-770 nm using a modified Karl Storz Image1 S^TM^ RUBINA^TM^ (KARL STORZ Endoskope, Tuttlingen, Germany) NIR and indocyanine green (ICG) endoscopic system. The system xenon light source (D-Light P, KARL STORZ Endoskope, Tuttlingen, Germany) provides both visibility and NIR excitation light after switching from white light to NIR mode. Dissection of all three cases proceeded with an ultrasonic scalpel. A single chest drain was placed and connected to a digital chest drainage system upon closing.

**Figure 1:**
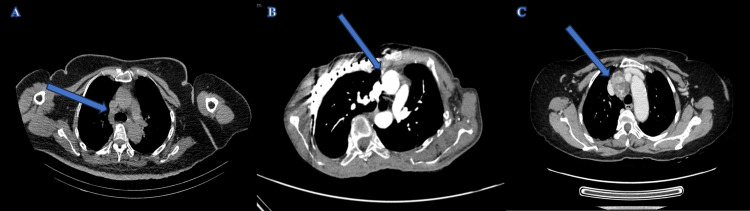
Computed tomography (CT) thorax of Case 1 (A) showed a hypodense lesion (arrow) at the 2R-4R region. Contrasted CT thorax of Case 2 (B) showed a hyperdense lesion (arrow) anterior to ascending aorta. Contrasted CT thorax of Case 3 (C) showed a heterogenous lesion (arrow) located between the right brachiocephalic vein and innominate artery.

**Figure 2:**
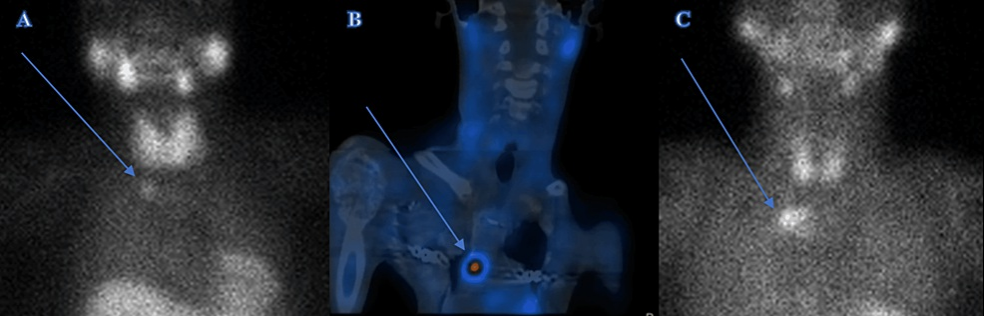
Tc99m-MIBI (methoxyisobutylisonitrile) scintigraphy of Case 1 (A), Case 3 (C), and single-photon emission computed tomography imaging (SPECT) scan of Case 2 (B) exhibited the mediastinal parathyroid adenomas (arrow).

There were three recruited patients: one male and two females. The mean age was 48.6 years old (range: 16-66 years). Clinical presentation with pre-operative and post-operative serum calcium and intact parathyroid hormone (iPTH) levels are shown in Table 1. Two patients with PHPT presented with fatigue and respiratory muscle weakness. Biochemical tests showed a mean serum calcium level of 3.52 mmol/L (range: 2.84-4.38 mmol/L) and a mean iPTH level of 274.6 pmol/L (range: 8.87-695 pmol/L). Computed tomography (CT) thorax reported a mass in the anterior mediastinal region in all patients with an average size of 2.4 x 2.1 x 2.3cm (range: 1.3-3.8cm) (Figure 1). 99mTc-hexakis-2-methoxyisobuthylisonitrile (Tc99m-MIBI) scintigraphy reported uptake in all patients (Figure 2).

**Table 1:** Clinical characteristics, perioperative, and histopathological findings of patients with primary hyperparathyroidism with mediastinal parathyroid lesion. iPTH: intact parathyroid hormone, VATS: video-assisted thoracoscopic surgery

Case	1	2	3	Mean ± SD
Preoperative findings				
Age (years)	64	16	66	48.67±26.15
Gender	Female	Male	Female	
Chief complaint	Fatigue	Incidental finding of hypercalcemia	Shortness of breath and fatigue	
Co-morbidities	Hypertension, diabetes melitus, bronchial asthma	Pectus excavatum (corrective surgery done)	Hypertension, Hashimoto's thyroiditis	
Calcium level (2.25-2.63mmol/L)	2.84	4.38	3.36	3.53±0.72
iPTH level (1.59-7.24 pmol/L)	8.87	695	120	274.62±340.26
Perioperative findings				
Location of tumors	Right lower paratracheal (2R, 4R region)	Anterior to ascending aorta	Between innominate artery and right brachiocephalic vein	
Size of tumours of CT thorax (cm)	1.3x1.3x1.5	2.2x1.8x2.0	3.8x3.2x3.4	2.4±1.1 x 2.1±0.9 x 2.3±0.9
Surgical approach	Uniportal VATS	Uniportal VATS	Partial sternotomy	
Duration of surgery (minutes)	80	170	125	125±41.58
Estimated blood loss (ml)	Minimal	100	200	100±92.40
Post-operative calcium level (mmol/L)	2.4	2.37	2.47	2.41±0.05
Post-operative iPTH level (pmol/L)	1.03	6.11	5.7	4.28±2.61
Post-operative complication	No	No	No	
Duration of stay (days)	4	5	14	7.66±5.09
Histopathological findings				
Pathology	Adenoma	Adenoma	Adenoma	

Uniportal VATS and parathyroidectomy were performed in all cases except in Case 3, whereby the surgery was performed through an upper partial sternotomy. In all of these cases, the MPA was successfully located and excised the MPA. The average operating time was 125 minutes with an average blood loss of 100ml. Post-operative serum calcium and iPTH normalized in all patients. The final histopathological results were conclusive of parathyroid adenoma. All three patients recovered without complication and no recurrence upon six months of follow-up.

## Discussion

PHPT is caused by hypersecretion of PTH resulting in hypercalcemia. Eighty-five percent of cases of PHPT are caused by single or multiple hyperfunctioning adenomas, 12-15% are due to parathyroid hyperplasia, and the remaining 1-3% are carcinoma [3]. Most parathyroid adenomas are typically situated in the neck adjacent to the thyroid gland. However, the remainder of ectopic adenomas might be located adjacent to the thymus, para esophageal spaces, or within the carotid sheath [4].

Patients with MPA often present with symptoms of hypercalcemia which could manifest as gastrointestinal discomfort, constipation, general malaise, urinary stones, bone lesions, or fractures (osteoporosis) [5]. The disease could occur as part of the clinical manifestation of multiple endocrine neoplasia type 1 (MEN1) or type 2a (MEN2a) [6].

Tc99m-MIBI scintigraphy remains the gold standard for preoperative imaging and has shown a sensitivity of 70-83.6% and specificity of 98.3% in the localization of parathyroid adenomas [7,8]. However, thymoma might mistakenly give a false positivity on the Tc99m-MIBI scan, whereas false negative imaging can be due to multiple adenomas or asymmetric hyperplasia [9]. In our series, all the ectopic adenomas were detected through the Tc99m-MIBI scan.

It has been reported that 2% of these ectopic MPA are difficult to be excised via a cervical incision [1,2]. Various surgical approaches for MPA have been described; median sternotomy or thoracotomy has been accepted as the standard approach. VATS was proven to be a practical and safe method with faster recovery time [10,11]. Infrequent complications such as recurrent laryngeal nerve injury, transient vocal cord palsy, and bleeding were reported by the VATS approach [12]. We did not see any such complications in our series.

Case No. 3 was suspicious of parathyroid carcinoma due to its size, CT scan appearance, serum calcium level, and with presence of multiple mediastinal lymphadenopathy [13]. Given the suspicion of carcinoma and the proximity of the tumor between the superior vena cava and ascending aorta led to an upper partial sternotomy and lymphadenectomy to ensure en-mass removal without tumor capsule disruption [14].

Several intraoperative adjuncts such as methylene blue (MB), near-infrared fluorescence (NIF), and infrared spectroscopy have been described to improve visualization of the parathyroid gland [15]. Intravenous MB is efficacious in parathyroid identification; however, its neurotoxicity is debatable [16]. An NIR camera or spectroscope can be used to detect autofluorescence (dye-free technique) which allows a precise real-time identification of the lesion [17]. The sensitivity and specificity are 94.1% and 80%, respectively [18]. Parathyroid adenoma is expected to show NIR autofluorescence in NIR mode and display a well-recognizable bluish-violet color on the monitor [15]. We utilized intraoperative NIR parathyroid gland identification by autofluorescence, a dye-free technique. Presently, ICG fluorescence angiography is widely used with its NIR camera system for localization. ICG is safe and feasible which enables instant localization of MPA especially in the VATS approach [19].

## Conclusions

MPA is a rare cause of PHPT. Comprehensive diagnostic imaging and surgical planning are important for the localization of MPA. Although various surgical approaches have been proposed, a uniportal VATS approach may provide faster recovery with less morbidity.
